# Central Nervous System Demyelination in a Charcot-Marie-Tooth Type 1A Patient

**DOI:** 10.1155/2013/243652

**Published:** 2013-12-16

**Authors:** Christos Koros, Maria-Eleftheria Evangelopoulos, Costas Kilidireas, Elisabeth Andreadou

**Affiliations:** 1st Department of Neurology, Athens National University, “Aeginition” Hospital, 74 Vas. Sophia's Avenue, 11528 Athens, Greece

## Abstract

*Introduction*. Central nervous system involvement, either clinical or subclinical, has been reported mainly in X-linked Charcot-Marie-Tooth (CMT-X) patients. *Case Presentation*. We present the case of a 31-year-old man with a genetically confirmed history of CMT1A who developed CNS involvement mimicking multiple sclerosis (MS). Clinical, imaging, and laboratory findings suggested an autoimmune CNS demyelination. *Discussion*. Although the simultaneous existence of CMT1A and MS could be coincidental we postulate that overexpression of PMP22, the target protein in CMT1A, might influence the immunological self-tolerance to CNS proteins via molecular mimicry, leading to a CNS autoimmune demyelinating disorder.

## 1. Introduction

Charcot-Marie-Tooth (CMT) disease represents a heterogeneous group of inherited neuropathies characterized by distal limb weakness and atrophy, sensory loss, and decreased or absent tendon reflexes. Despite the fact that the core symptoms of CMT involve the peripheral nervous system (PNS), central nervous system (CNS) involvement either in the form of clinical symptoms or magnetic resonance imaging (MRI) white matter lesions has been occasionally reported, mainly for the X-linked type of CMT [1, 2].

## 2. Case Presentation

Herein, we present the case of a 31-year-old man with a history of CMT1A who developed CNS involvement mimicking multiple sclerosis.

During his army duty, five years before presentation, he complained of gait disorder. Bilateral atrophy of the distal lower extremities was observed. Electrophysiological investigation revealed slow motor and sensory nerve conduction velocities in upper and lower limbs indicative of a predominantly demyelinating polyneuropathy with secondary axonal loss (Table 1). The CMT1A diagnosis was confirmed, by means of molecular evaluation [peripheral myelin protein 22 gene (PMP22) duplication]. However, his family history was negative.

The patient was initially referred to us because of a 3-day history of diplopia, present in horizontal gaze positions. He also reported an episode of right hemibody dysesthesias two weeks earlier, with spontaneous remission within three days.

Clinical examination revealed distal muscle weakness and atrophy more pronounced in the lower limbs, pes cavus, galloping gait, absent tendon reflexes, distal hypoesthesia in the lower limbs, and decreased sensation to vibration in the toes. There was no evidence of nerve hypertrophy or fasciculations. Examination of the cranial nerves showed a right intranuclear ophthalmoplegia with dissociative nystagmus along with right abducens nerve paresis.

**Figure 1:**
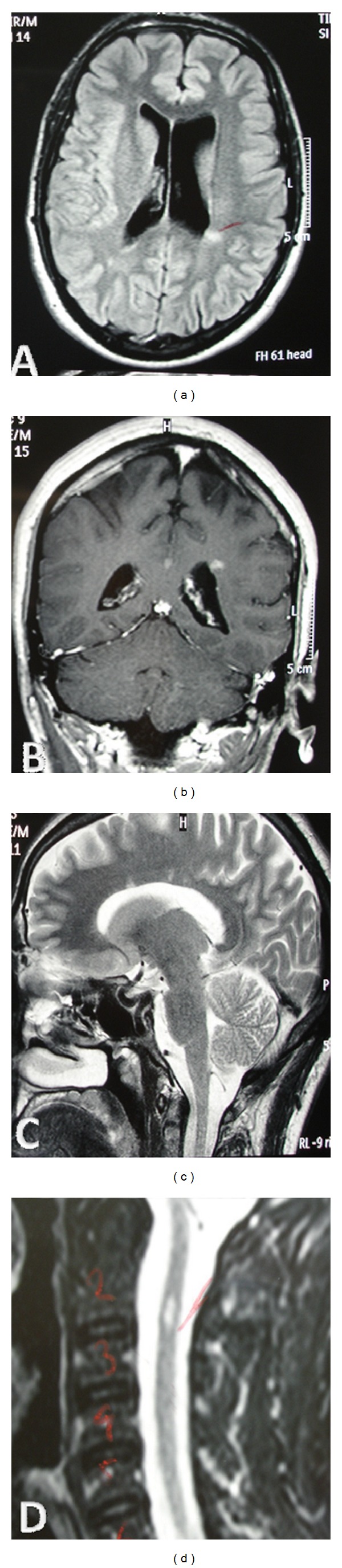
Magnetic resonance imaging of the brain and spinal cord at first attack, showing lesions indicative of multiple sclerosis. (a) Axial FLAIR image showing hyperintense lesions in the white matter of both hemispheres. (b) Coronal T1-weighted image showing a gadolinium-enhancing lesion near the left lateral ventricle. (c) Sagittal T2-weighted image showing lesions in the corpus callosum. (d) Sagittal T2-weighted image showing a hyperintense lesion on cervical spinal cord at C2 level.

Blood investigations including serum vitamin B12 level and thyroid hormones were normal. HIV, syphilis, and antinuclear antibody titers were negative. Cerebrospinal fluid (CSF) studies showed a mild increase in protein level (52 mg/dL, normal <45 mg/dL) and a marginal IgG index (0.65 normal <0.65), without pleocytosis or oligoclonal bands. Electrophysiological evaluation did not reveal any changes compared to the initial one, neither conduction block nor temporal dispersion of compound muscle action potentials (Table 1). Brain MRI fulfilled the Barkhof's criteria for MS diagnosis with several periventricular, subcortical, and callosal hyperintense lesions on T2-weighted and FLAIR images (Figures 1(a) and 1(c)), one of which (in the periventricular white matter of the left hemisphere) showed gadolinium-enhancement on T1-weighted sequences (Figure 1(b)). Additionally, cervical spinal cord MRI revealed a lesion at C2 level (Figure 1(d)).

**Figure 2:**
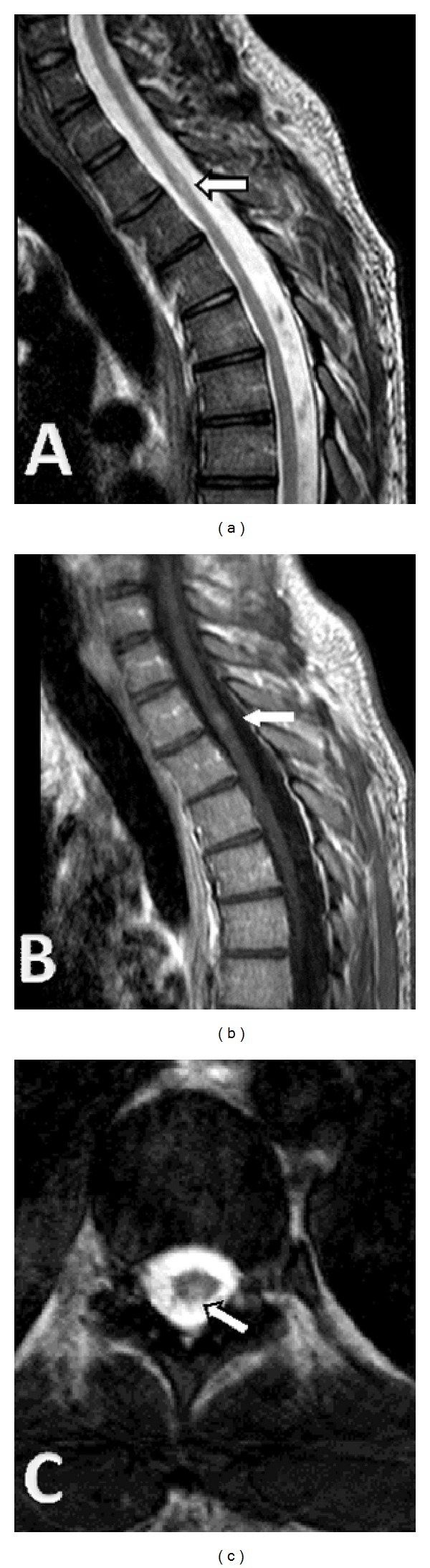
Thoracic spinal cord MRI at second attack. (a) Sagittal T2-weighted image showing a hyperintense lesion at T3 level. (b) Sagittal T1-weighted image showing gadolinium-enhancement of the lesion at T3 level. (c) Axial T2-weighted image showing a hyperintense lesion at T11 level.

The patient was treated with high-dose intravenous methylprednisolone (1 g daily for five consecutive days) followed by an oral taper. Complete remission of diplopia was achieved within 2 weeks. Ten months later, he had a second attack characterized by weakness of the distal part of the right lower limb. Brain MRI did not demonstrate significant alterations as compared to the previous one. However, spinal cord MRI revealed one gadolinium-enhancing lesion in the thoracic segment at T3 level along with a nonenhancing lesion at T11 level (Figure 2). Corticosteroid therapy was applied with complete remission within 5-6 days. Subsequently, the patient was started on immunomodulatory treatment with glatiramer acetate. In the following months he had three mild relapses consisting of left lower limb paresis, diplopia, and left optic neuritis, respectively, without residual deficits. A follow-up brain MRI at six months after treatment revealed two new nonenhancing lesions in the right temporal lobe (a periventricular and a subcortical one, resp.). Three months later, the patient had another relapse consisting of right optic neuritis. Thereafter, switching to fingolimod was decided. Approximately six months after initiation of treatment, the patient is free of relapses and fingolimod remains well tolerated. Long-term tolerance and effectiveness of the treatment remain to be evaluated.

## 3. Discussion

Our patient, with a confirmed history of CMT1A [3], developed symptoms typical of relapsing multiple sclerosis. Additionally, brain and spinal cord MRI demonstrated white matter lesions consistent with MS. Although an incidental coexistence of MS and CMT-1A in the presented case could not be excluded, the possibility of a causal association could be hypothesized.

Concomitant central and peripheral demyelination represents a relatively rare clinical entity. In most cases it can be attributed to an autoimmune inflammatory process affecting both the CNS and PNS. Occasionally, these combined disorders fulfill the criteria for MS and/or chronic inflammatory demyelinating polyradiculoneuropathy (CIDP) [4]. A recent study reported specific antineurofascin antibody immunoreactivity in patients with combined central and peripheral demyelination [5].

Interestingly, CNS involvement has been also observed in inherited neuropathies including various subtypes of CMT [6], the most common of which is the X-linked form of the disease [1, 2]. The patterns of abnormal central function range from asymptomatic transient white matter lesions to deafness and motor or sensory symptoms. The observation that connexin 32 (Cx32), the target molecule in X-linked CMT, is expressed in both the CNS and PNS may account for the presence of lesions in the CNS. Evidence of concomitant demyelination in the central and peripheral nervous system is scarce in other subtypes of CMT. As far as CMT1A is concerned, central symptoms were present in a limited number of literature reports [7–10]. Frasson and coauthors described two cases of genetically confirmed CMT1A with duplication of PMP22 gene that also developed clinically definite multiple sclerosis [7].

These observations raise the possibility of a causal relationship between the two conditions in our patient, probably through an immunological mechanism. There is evidence to suggest that PMP22, the target protein in CMT1A—despite being considered to be expressed selectively in the periphery—shares partial homology with other CNS proteins like the proteolipid protein (PLP) [7]. Therefore, it could be hypothesized that PMP22 overexpression might influence the immunological self-tolerance to CNS proteins via molecular mimicry, leading to a CNS autoimmune demyelinating disorder. In a recent case report of a CMT1A patient exhibiting recurrent optic neuritis episodes, the authors highlight the putative role of PMP22 overexpression in the development of CNS inflammation, by characterizing peripheral T-cell responses to a panel of myelin epitopes expressed in the CNS [10]. Finally, experimental data supporting PMP22 expression by selective CNS neurons (including spinal cord and brainstem ones) in human might implicate a direct CNS autoimmune pathway against PMP22 in CMT1A patients [11]. Given the rarity of central involvement in CMT1A, its nature remains elusive. Should a causal association between CNS and PNS lesions be established, possible pathogenetic mechanisms could be hypothesized.

Briefly, although the simultaneous existence of CMT1A and MS in our patient could be coincidental, we postulate that overexpression of PMP22, the target protein in CMT1A, might have influenced the immunological self-tolerance to CNS proteins via molecular mimicry and led to CNS demyelination.

## Figures and Tables

**Table 1:** Comparative motor and sensory conduction studies of the patient at diagnosis of CMT and during present evaluation.

Conduction studies^†^			
Nerve			
Motor conduction	Conduction velocity (m/s)	Amplitude (mV)	Latency (ms)
Median R	36.5/36	3.5/4.5	5.8/5.0
Ulnar R	38/42	5.0/3.5	3.9/3.4
Peroneal R	23/ND	0.2/ND	8.8/ND
Tibial R	30/27	0.5/0.28	9.2/9.8

Sensory conduction	Conduction velocity (m/s)	Amplitude (*μ*V)	Latency (ms)

Median R	38/42	1.5/1.9	4.7/4.3
Ulnar R	39/ND	2/ND	4.2/ND
Sural R	36/32	2.5/2.2	2.5/3.9
Peroneal R	Could not be elicited		

^†^At diagnosis of CMT/during present evaluation.

ND: not done.
